# Dual Role of cAMP in the Transcriptional Regulation of Multidrug Resistance-Associated Protein 4 (MRP4) in Pancreatic Adenocarcinoma Cell Lines

**DOI:** 10.1371/journal.pone.0120651

**Published:** 2015-03-19

**Authors:** Alejandro Carozzo, Federico Diez, Natalia Gomez, Maia Cabrera, Carina Shayo, Carlos Davio, Natalia Fernández

**Affiliations:** 1 Laboratorio de Farmacología de Receptores, Cátedra de Química Medicinal, Departamento de Farmacología, Facultad de Farmacia y Bioquímica, Universidad de Buenos Aires, Buenos Aires, Argentina; 2 Laboratorio de Patología y Farmacología Molecular, Instituto de Biología y Medicina Experimental, Buenos Aires, Argentina; 3 Consejo Nacional de Investigaciones Científicas y Técnicas, Buenos Aires, Argentina; University of Texas Health Science Center at Houston, UNITED STATES

## Abstract

Cyclic AMP represents one of the most studied signaling molecules and its role in proliferation and differentiation processes has been well established. Intracellular cAMP levels are tightly regulated where the MRP4 transporter plays a major role. In the present study, we sought to establish whether cAMP modulated MRP4 expression in pancreatic adenocarcinoma cell lines. Quantitative PCR and western blot studies showed that cAMP-increasing agents enhanced MRP4 transcripts and protein levels in PANC-1 cells. Reporter luciferase experiments carried out in pancreatic AR42J cells showed that intracellular cAMP up-regulates MRP4 through an Epac2- and Rap1- mediated mechanism whereas extracellular cAMP reduced MRP4 promoter activity by a MEK/ERK-mediated pathway. Present results show that cAMP regulates MRP4 promoter activity, and further indicate that the balance between intracellular and extracellular cAMP levels determines MRP4 expression.

## Introduction

Cyclic AMP (cAMP), the first second messenger discovered, is one of the most studied signaling molecules and plays a critical role in cellular responses to extracellular stimuli. It controls a wide spectrum of biological effects including cell proliferation, differentiation, and apoptosis. The intracellular regulation of cAMP depends on the balance between its production by adenylyl cyclase, an enzyme stimulated by diverse hormones and neurotransmitters, and its degradation by phosphodiesterases (PDEs) [1]. However, recent studies support that the efflux of cAMP through members of the multidrug associated resistance protein family (MRPs) like MRP4, MRP5, and MRP8, constitutes an additional relevant mechanism involved in the regulation of cAMP signaling [2–5].

The MRP family belonging to the ATP-binding cassette (ABC) subfamily C of transporters participates not only in the development of resistance to chemotherapeutic agents but also in the efflux of various molecules involved in cellular physiology [6]. Therefore, the major role of these transporters is the efflux of drugs and endogenous molecules.

In particular, MRP4 is ubiquitously expressed, showing high expression levels in the prostate, liver, kidney, brain, as well as in hematopoietic cells [4,7–9]. Substrates for MRP4 include antineoplasic drugs (methotrexate, 6-mercaptopurine, 6-thioguanine), antiviral agents (azidothymidine, tenofovir, lamivudine, and ganciclovir) and endogenous molecules (prostaglandins, cyclic nucleotides, steroids, bile acids and folate) [10–13].

Current evidence supports that MRP4 plays a dual role in cancer given that it not only mediates the efflux of chemotherapeutic agents but it also transports prostaglandins and cyclic nucleotides that are involved in the regulation of cell proliferation and differentiation.

We previously reported that MRPs, in particular MRP4, play a relevant role in intracellular cAMP regulation in human acute myeloid leukemia cells [14]. This study provided the first experimental evidence that MRP4 and cAMP extrusion may represent a new potential target for differentiation therapy. Moreover, several clinical studies using tumor genome sequencing showed MRP4 expression as a prognosis marker in esophageal [15], gastric [16], rectal [17], lung [18], ovarian [19] and prostate [20] cancer as well as in neuroblastoma [21]. Furthermore, current evidence also supports that MRP4 expression may represent a relevant pharmacological target [22].

Pancreatic ductal adenocarcinoma (PDAC) is the fourth leading cause of cancer related death in the world but it is projected to be the second cause by 2030 [23]. Its poor prognosis is attributable mainly to the lack of early detection methods and effective treatments [24]. The resistance of PDAC to various forms of chemotherapy has been associated to both intrinsic and/or acquired MRPs-mediated resistance in these cancer cells. Recent studies show that MRP4 expression is higher in PDAC than in normal pancreatic tissue [25]. MRP4 regulation has not been extensively studied, but recent reports show the importance of xenobiotics in MRP4 regulation at the transcriptional level in the liver [26].

Given the importance of MRP4 in the regulation of intracellular cAMP levels and the limited knowledge regarding its regulation, the aim of the present work was to evaluate the role of cAMP in MRP4 expression as well as the underlying signaling pathways. Present findings show that cAMP regulates the activity of MRP4 promoter through a dual mechanism. While intracellular cAMP (i-cAMP) induces MRP4 expression through an Epac2/Rap1 mechanism, extracellular cAMP (e-cAMP) inhibits MRP4 promoter activity by a MEK/ERK pathway. Considering the relevance of cAMP tight regulation in cell proliferation and differentiation, it is possible to hypothesize that this mechanism may compensate high and sustained increases in i-cAMP levels.

## Materials and Methods

### Materials

RPMI-1640 medium, DMEM medium, antibiotics, phosphate-buffered saline (PBS), bovine serum albumin (BSA), 3-isobutyl-1-methylxanthine (IBMX), cAMP, db-cAMP, forskolin, PGE2, KT5720, cycloheximide and ESI-09 were obtained from Sigma. Fetal bovine serum (FBS) was purchased from Natocor. MK-571 (3-([(3-(2-[7-chloro-2-quinolinyl]ethenyl)phenyl)-([3-dimethylamino-3-oxopropyl)-thio)-methyl]thio) propanoic acid) was obtained from Calbiochem. 8-(4-Chlorophenylthio)-2'-O-methyladenosine-3',5'-cyclic monophosphate (8-CPT-2Me-cAMP) was from Tocris Bioscience. [^3^H]cAMP was purchased from PerkinElmer Life Sciences. All other chemicals were of analytical grade and obtained from standard sources.

### Cell culture

PANC-1 (ATCC, Rockville, MD, USA), an epithelial cell line derived from a human pancreatic adenocarcinoma of ductal cell origin, and AR42J cells (ATCC, Rockville, MD, USA) derived from a rat pancreatic tumor were grown in 25cm^2^ flasks at 37°C in a humidified 5% CO_2_ atmosphere in RPMI-1640 medium and DMEM medium respectively, supplemented with 10% fetal bovine serum and 50μg/mL gentamicin.

### cAMP Assay

AR42J cells were seeded in 48-well plates in DMEM medium at a density of 1x10^5^ cells/well and exposed to various agents at different concentrations and time points as indicated in the corresponding figure legends. Following treatment, supernatants were removed and placed over 0.8ml of ethanol (extracellular cAMP) and 0.8ml of ethanol was added to each well (intracellular cAMP). Ethanol was dried out, and residues suspended in 50mM Tris-HCl, pH 7.4, 0.1% BSA for further cAMP determination. Cyclic AMP content was determined by a competitive radio-binding assay for PKA using [^3^H]-cAMP as previously described [27]. The standard curve was performed using eight cAMP concentrations ranging from 0.1 to 90 pmol. Duplicate samples in at least three independent experiments were analyzed.

### RT-PCR and Quantitative Real-time PCR

Total RNA was isolated from PANC-1 cells using Quick-Zol reagent (Kalium Technologies) following the manufacturer’s instructions. For the first-strand cDNA synthesis, 1μg of total RNA was reverse-transcribed using the High Capacity cDNA Reverse Transcription kit (AB) with random primers. Quantitative real-time PCR (qPCR) was performed using 1μL of the resulting cDNA, amplified at 45 cycles for 15s at 94°C, 20s at melting temperature (60°C), and 30s at 72°C using the HOT FIREPol EvaGreen qPCR Mix Plus (Solis Biodyne). Quantitative PCR was performed in triplicate using the Rotor Gene Q detection system (Qiagen) and the following primers: human MRP4 forward, 5’-GGACAAAGACAACTGGTGTGCC-3’ and reverse, 5’-AATGGTTAGCACGGTGCAGTGG-3’; and human β-Actin (βAct) forward, 5’-GGACTTCGAGCAAGAGATGG-3’ and reverse 5’-AGCACTGTGTTGGCGTACAG-3’. The specificity of each primer set was monitored by analyzing the dissociation curve, and the relative MRP4 mRNA quantification was performed using the comparative ΔΔCt method using Actin as the housekeeping gene.

### Plasmid constructs

pCMV-Myc-EPAC, pCMV-Myc-N-EPAC, pMT2-HA-EPAC2, pCGN-HA-Rap1a, pCGN-HA-Rap1b and pMT2—HA-Rap1GAP plasmids [28] were kindly provided by Dr. Omar Coso (Department of Physiology and Molecular Biology, FCEN, UBA, Buenos Aires, Argentina). pGFP-PKI, pGFP-PKImut, pcDNA3-DN-HRas and pcDNA3-DN-KRas plasmids were a kind gift of Dr J. Silvio Gutkind (Oral and Pharyngeal Cancer Branch, National Institutes of Health, Bethesda, USA). pACL4-AKT1-Δ4-129 (AKTmyr) was a gift from Dr. Virgina Novaro (Laboratorio de Carcinogénesis Hormonal, IBYME-CONICET, Buenos Aires, Argentina).

A 2372bp fraction of the 5’-flanking region of the human MRP4 gene was amplified using the RP11-789G22 BAC clone as template (license provided by the Sanger Institute, Hinxton, UK) and the following primers: forward (+48bp-BglII-fw) 5’-CGCAGATCTACCTCAAGCAGGGATG-3’ and reverse (-2324bp-KpnI-rv) 5’-CTGGTACCGCTGGGATTATGGGCTTG-3’ (positions relative to translational starting site). The amplification product was cloned into the pGL3-basic (Promega) firefly luciferase reporter vector (named MRP4-Luc) and the integrity of the sequence was controlled by sequencing.

### Transfection and reporter gene assays

AR42J cells seeded on 12-well plates were transfected using the K2 Transfection System (Biontex, Munich, Germany) with the pMRP4-Luc luciferase reporter plasmid according to the manufacturer's instructions. In some experiments, cells were also co-transfected with the plasmid constructs previously detailed or an empty vector to maintain the total amount of DNA equal. After 6h, cells were seeded in 96-well plates and stimulated with diverse agents after 24h. Luciferase activity was measured 24h later with the Steady-Glo Luciferase Assay System according to the manufacturer's instructions (Promega Biosciences Inc. San Luis Obispo, CA, USA) using the FlexStation 3 Multi-Mode Microplate Reader (Molecular Devices, LLC). Experimental reporter activity was normalized by the CellTiter 96 AQueous One Solution Cell Proliferation Assay (MTS) kit (Promega) and the data was expressed as a percent of each basal condition.

### Western Blot Assay

Cells were lysed in 50mM Tris-HCl pH 6.8, 2% SDS, 100mM 2-mercaptoethanol, 10% glycerol, and 0.05% bromophenol blue and sonicated to shear DNA. Total cell lysates were resolved by 8% SDS-PAGE for Multidrug-Resistance Protein 4 (MRP4) detection or 10% SDS PAGE for pERK detection, blotted, and incubated with 3μg/ml rat monoclonal anti-MRP4 M4I-10 (Alexis Biochemicals); 1μg/ml mouse monoclonal anti-pERK or ERK1/2 (Santa Cruz Biotechnology); or 1μg/ml rabbit anti-actin antibody (Santa Cruz Biotechnology) in PBS containing 0.05% Tween 20. All subsequent washes were performed with the same buffer. Reactivity was developed using an anti-rat, anti-mouse, or anti-rabbit polyclonal antibody linked to horseradish peroxidase (Santa Cruz Biotechnology) and enhanced chemiluminescence reagents following the manufacturer’s instructions (Amersham Biosciences).

### Data Analysis

Results are expressed as mean ± SEM. Statistical analysis was performed by one-way analysis of variance followed by Bonferroni’s multiple comparison test. Values of p<0.05 were considered statistically significant.

## Results and Discussion

**Fig 1:**
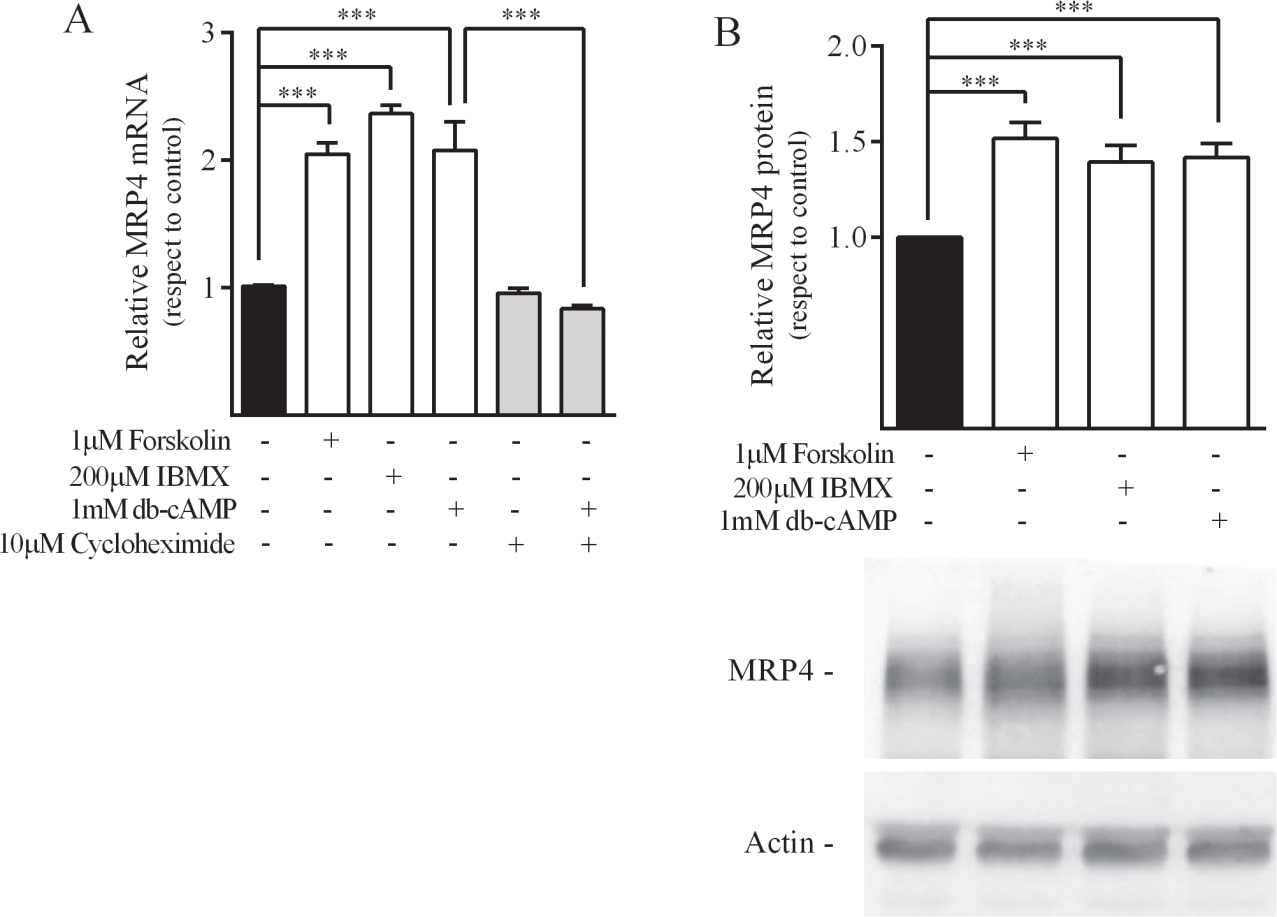
Effect of cAMP modulating agents on MRP4 expression in PANC-1 cells. Cells were exposed to agents that modulate the cAMP pathway at the indicated concentrations. Cells were harvested 24h after stimulation for mRNA quantitation, and after 48h for protein detection by immunoblotting. ***A*.** MRP4 mRNA was quantified by real-time PCR, normalized by β-actin mRNA and expressed relative to control (mean±SD; n = 3). ***B*.** *top*. Densitometric quantification of MRP4 protein bands normalized to β-actin and expressed relative to control (mean±SD; n = 6), *bottom*, representative western blot assay of six independent experiments is shown. *** p< 0.001.

In order to evaluate the role of cAMP in modulating MRP4 expression in pancreatic cell lines, MRP4 mRNA transcripts were assessed by real-time PCR. PANC-1 cells, a widely used epithelial ductal pancreatic carcinoma model, were exposed to forskolin (adenylyl cyclase direct activator), IBMX (PDE inhibitor) and db-cAMP (cAMP permeable analog). Following cell exposure, all the agents assessed led to an approximate 2-fold increase in MRP4 mRNA levels as compared with untreated cells. These results indicate that cAMP increasing agents up-regulate MRP4. It is worth noting that cycloheximide treatment abolished db-cAMP-stimulated increase in MRP4 mRNA, suggesting that *de novo* synthesis is required for cAMP-mediated MRP4 transcription (Fig. 1A). In accordance, when MRP4 protein levels were analyzed by western blot, we observed a significant increase in MRP4 expression in cells exposed to forskolin, db-cAMP and IBMX (Fig. 1B). The increase in both mRNA and protein levels may result from a higher stability in mRNA transcripts as well as by MRP4 transcription stimulation. Therefore, we addressed MRP4 promoter activity by luciferase reporter assays.

In an attempt to study MRP4 transcriptional regulation we analyzed a 2.5kb region upstream to the transcriptional start site (TSS) of the human MRP4 gene using the Mat Inspector software (Genomatrix Suite Software). The *in silico* analysis revealed three putative CREB protein binding sites. In addition, other sites related to pathways that crosstalk with cAMP signaling like ELK1, AP1 and Myc were also identified (S1A Fig.). Therefore, we cloned a 2372bp fragment from the human MRP4 gene from +48 to −2324bp respect to the TSS into the pGL3-basic reporter vector (named MRP4-Luc) and assayed luciferase activity in another pancreatic model, AR42J cells [29], where the expression levels of MRP4 are lower than in PANC-1cells, facilitating the study of promoter activating agents, and where transfection and luciferase assays are extensively performed. Luciferase reporter assay showed that this construct led to a 45-fold higher activity than the empty vector (S1B Fig.) indicating a significant constitutive activity of the promoter in this system.

**Fig 2:**
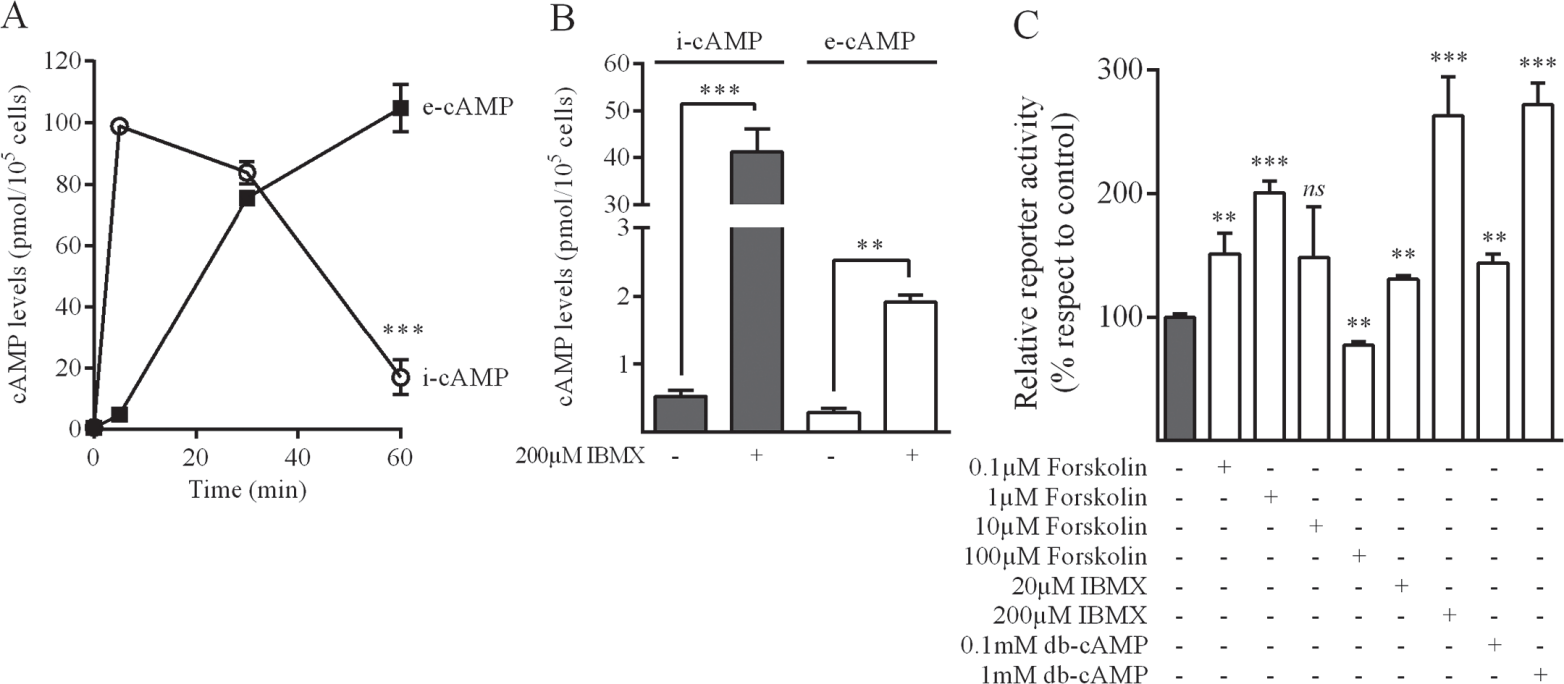
Effect of cAMP modulating agents on MRP4 promoter activity in AR42J cells. AR42J cells were stimulated during the indicated times with 1μM forskolin *(* ***A***) or with 200μM IBMX for 15 min (***B***), and cAMP levels were determined as detailed under Materials and Methods. ***C*.** Cells were transfected with MRP4-Luc and treated with different agents as indicated. Luciferase activity was measured after 24h and data was referred to control. (mean±SD; n = 3), **p<0.01, ***p<0.001.

We next evaluated cAMP response in this system by kinetic experiments. Treatment with 1μM forskolin showed a rapid increase in intracellular cAMP levels that was followed by a delayed increment in extracellular levels. It is worth noting that even after 60 min of treatment with forskolin, i-cAMP concentration was still significantly different from basal level (Fig. 2A). In the same way, 15 min treatment with IBMX also increased i-cAMP from 0.5±0.1 to 41±5 pmol/well, while e-cAMP increased from 0.30±0.05 to 1.9±0.1 pmol/well (Fig. 2B). These results indicate that a generalized and sustained increase in i-cAMP levels is achieved with forskolin and IBMX, and that cAMP is extruded in both basal and stimulated conditions. When luciferase activity was evaluated in MRP4-Luc transfected AR42J cells following exposure to cAMP modulating agents, it was observed that both db-cAMP and IBMX caused a concentration-dependent increase in luciferase activity. Interestingly, the treatment with low concentrations of forskolin (0.1 and 1μM) also led to an increased in the reporter activity while the treatment with 100μM forskolin had the opposite effect (Fig. 2C).

These results indicate that the increment in cAMP levels achieved by the assayed agents induce MRP4 promoter activity. In this sense, it has been recently reported the existence of activating mutations in Gs proteins or GPCRs which lead to exacerbated cAMP signaling in diverse tumors including pancreatic carcinoma [30]. Based on these findings and present results, the higher expression of MRP4 observed in pancreatic ductal adenocarcinoma samples may be attributable to an increased and sustained cAMP production caused by mutations in Gs signaling.

**Fig 3:**
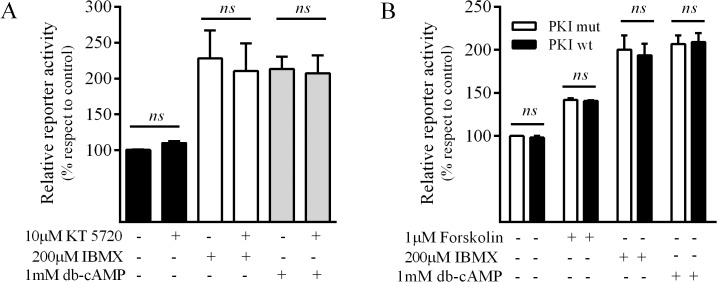
PKA pathway and regulation of MRP4 promoter activity in AR42J cells. Cells were transfected with MRP4-Luc and co-transfected using the constructs indicated. 24h after transfection the cells were treated with the different agents indicated. Luciferase activity was measured after 24h in cell lysates and data was referred to control. ***A*.** Effect of KT5720, a PKA pharmacological inhibitor, on db-cAMP- or IBMX-induced promoter activity (mean±SD; n = 3). ***B*.** Effect of the co-transfection with PKI *wt* (wild type protein kinase inhibitor) or PKI *mut* (inactive protein kinase inhibitor mutant) on db-cAMP- or IBMX-induced promoter activity (mean±SD; n = 3). *ns*, non-significant differences.

Given that the major intracellular target for cAMP is protein kinase A (PKA) we next addressed whether this kinase mediated cAMP-induced MRP4 up-regulation. The widespread used PKA inhibitor, KT5720, failed to inhibit both db-cAMP and IBMX responses (Fig. 3A). These findings were further confirmed by the observation that the MRP4 promoter activity was neither modified by PKI, a PKA peptide inhibitor (PKI wt), nor by an inactive mutant of this peptide (PKI mut) used as control (Fig. 3B). Both KT5720 and PKI construct were able to inhibit the phosphorylation of PKA substrates in AR42J cells when evaluated by western blot using an anti-PKA phosphorylated substrates-antibody (data not shown). These findings indicate that the cAMP-mediated MRP4 promoter induction is independent of the PKA signaling in AR42J cells.

**Fig 4:**
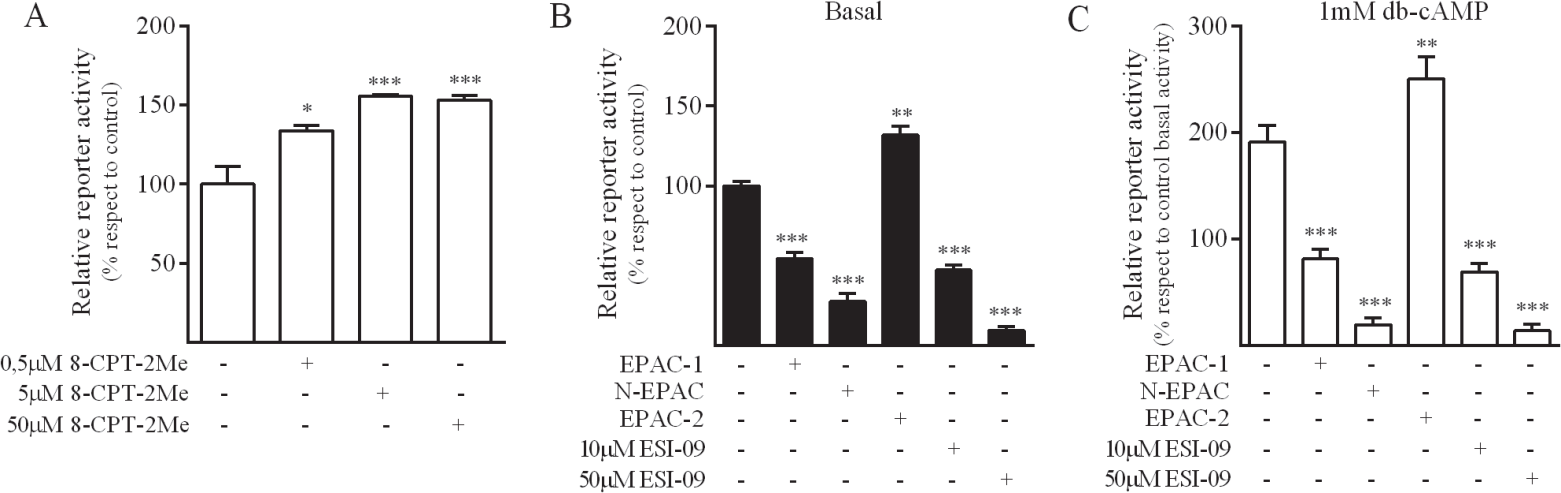
EPAC pathway and regulation of MRP4 promoter activity in AR42J cells. ***A*.** Cells were transfected with the MRP4-Luc plasmid and treated for 24h with 8-CPT-2Me-cAMP (8-CPT-2Me), a selective EPAC agonist, at the concentrations indicated (mean±SD; n = 2). ***B*, *C*.** AR42J cells were transfected with the MRP4-Luc plasmid and co-transfected with Epac1 (cAMP-GEFI), Epac2 (cAMP-GEFII) or N-Epac (dominant negative mutant) or pre-treated for 24h with ESI-09 (membrane-permeant inhibitor of Epac1 and Epac2). ***B*** corresponds to basal and ***C*** corresponds to db-cAMP stimulated promoter activity (mean±SD; n = 3). *p<0,05; **p<0.01; ***p<0.001.

Since PKA signaling did not mediate cAMP induction of MRP4, we next evaluated whether the exchange proteins directly activated by cAMP (Epacs) were involved in the regulation of MRP4 promoter by treating the cells with 8-CPT-2Me-cAMP, a selective activator of Epac. Results showed that 8-CPT-2Me-cAMP stimulated promoter activity in a concentration dependent fashion (Fig. 4A). Similarly, the co-transfection with Epac2, which contains an additional low affinity cAMP binding domain (CBD) [31,32], increased the promoter basal activity and further enhanced db-cAMP response (Fig. 4B and C). To confirm this observation, we co-transfected AR42J cells with a dominant negative mutant of Epac (N-Epac), that has been shown to abolish Epac GEF activity by competition. Consistently, N-Epac blunted db-cAMP response and reduced basal luciferase activity in AR42J cells; similar results were obtained with the Epac pharmacological inhibitor ESI-09 (Fig. 4B and C). Interestingly, Epac1 overexpression, behaved as the N-Epac construct, this could be explained in terms of competition for cAMP by Epac1 and Epac2, where only Epac2 is responsible of MRP4 promoter induction. Similar findings, regarding induction of MRP4 following cAMP sustained stimulation, were recently reported by other authors [33].

**Fig 5:**
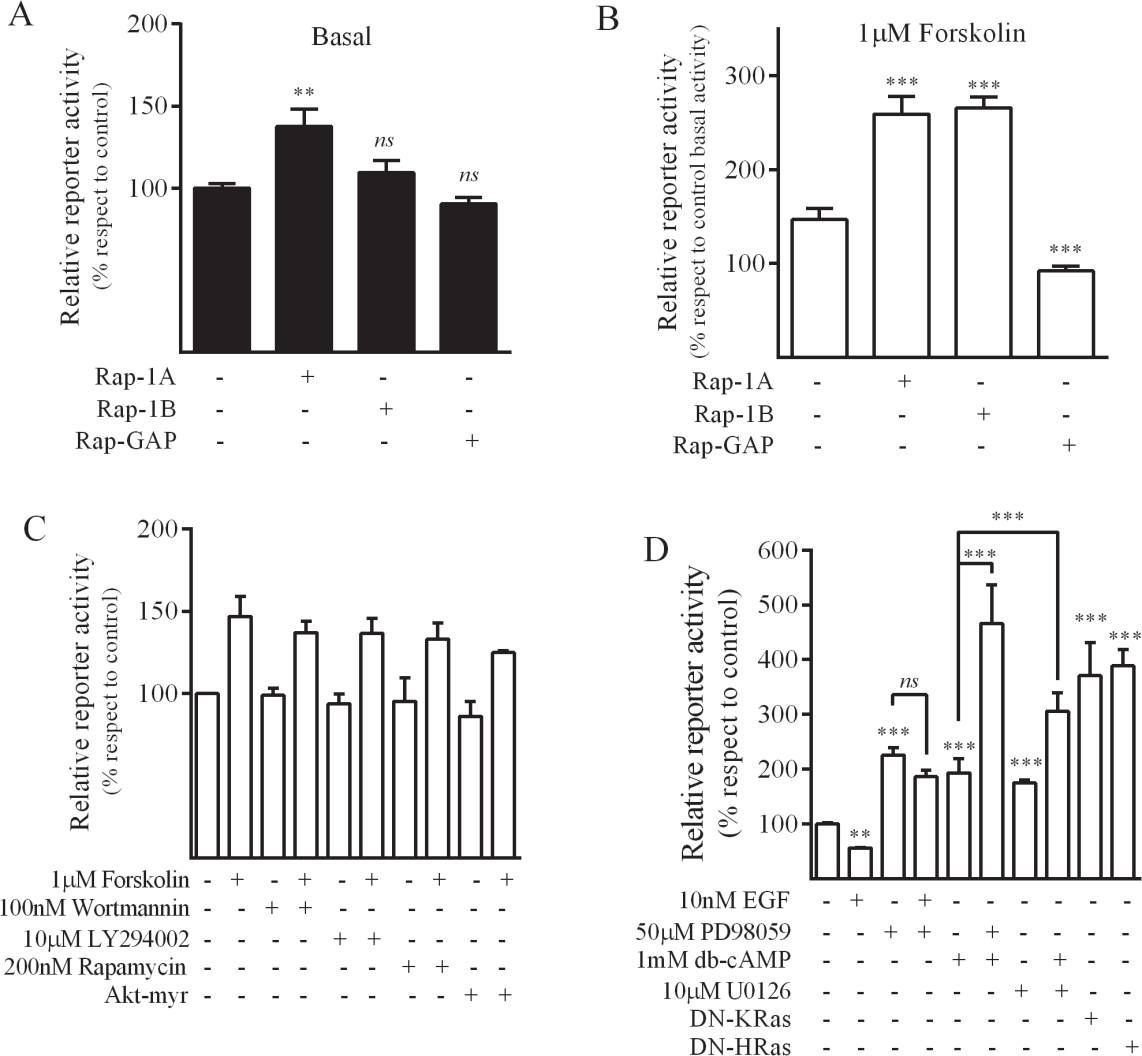
Signaling pathways involved on MRP4 promoter activity in AR42J cells. Cells were transfected with the MRP4-Luc plasmid and co-transfected with the constructs indicated. After 24h exposure to the indicated treatments, luciferase activities were measured in cell lysates; data was referred to control. ***A*, *B*.** *Rap involvement*. Cells were cotransfected with Rap1a, Rap1b or Rap1-GAP. ***A*** corresponds to basal and ***B*** corresponds to 1μM forskolin-stimulated promoter activity. (mean±SD; n = 3). ***C*.** *PI3K-Akt involvement*. Cells were cotransfected with Akt-myr, or treated with Wortmannin, LY294002 (PI3K inhibitors), or Rapamycine (mTOR kinase inhibitor) and forskolin-induced promoter activity was evaluated. (mean±SD; n = 3). ***D*.** *MEK-ERK involvement*. Cells were cotransfected with a Ras dominant negative mutant (DN-KRas or DN-HRas) or treated with EGF, PD985809, UO126 and/or db-cAMP and promoter activity was evaluated. (mean±SD; n = 3). **p<0.01; ***p<0.001; *ns*, non-significant differences.

Given that Epac proteins behave as specific GTP exchange factors (GEFs) for the Ras GTPase family members of Rap, we evaluated their downstream participation in Epac-mediated cAMP response. While only Rap1a significantly increased MRP4 basal promoter activity (Fig. 5A), both Rap1a and Rap1b enhanced forskolin-induced MRP4 promoter activity (Fig. 5B). Consistently, the Rap1GAP construct, which accelerates Rap1 GTP hydrolysis, abolished the effect of the cAMP response over the MRP4 promoter (Fig. 5B).

It has been previously described that PI3K is involved in the regulation of MRP4 expression by interleukin-6 [34] in keratinocytes and dermal fibroblasts. To further elucidate whether this signaling pathway is involved in cAMP-mediated induction of MRP4, inhibitors of the PI3K/Akt/mTOR pathway were used. Neither PI3K inhibitors (Wortmanin and LY294002) nor the mTOR inhibitor, Rapamycin, modified basal or induced MRP4 promoter activity. In accordance, the constitutive active form of Akt (Akt-myr) failed to modify luciferase activity (Fig. 5C) further supporting that the PI3K/Akt/mTOR pathway is not involved in cAMP mediated up-regulation of MRP4 in this model. Conversely, when MEK/ERK pathway was evaluated we observed that PD98059 and UO126 (MEK/ERK inhibitors) significantly enhanced both basal and db-cAMP-induced promoter activity. Furthermore, when we co-transfected AR42J cells with dominant negative mutants of the MEK/ERK upstream activators, K-Ras and H-Ras, we observed an increase in basal luciferase activity. In accordance, EGF treatment diminished the promoter activity, being this response inhibited by PD98059 (Fig. 5D). These findings indicate that in this system, the MEK/ERK pathway negatively modulates the MRP4 promoter activity. Taking into account that it has been previously reported that Rap1 antagonizes Ras activation and consequently abrogates MEK/ERK pathway [35], our results could be explained in terms of cAMP-mediated activation of Rap1. In addition, there is also evidence for the transcriptional activation of MAP kinase phosphatases (MKP1) by cAMP [36], which may explain not only cAMP-mediated MRP4 promoter regulation but also the observation that activation of MRP4 transcription by cAMP depends on *de novo* synthesis.

**Fig 6:**
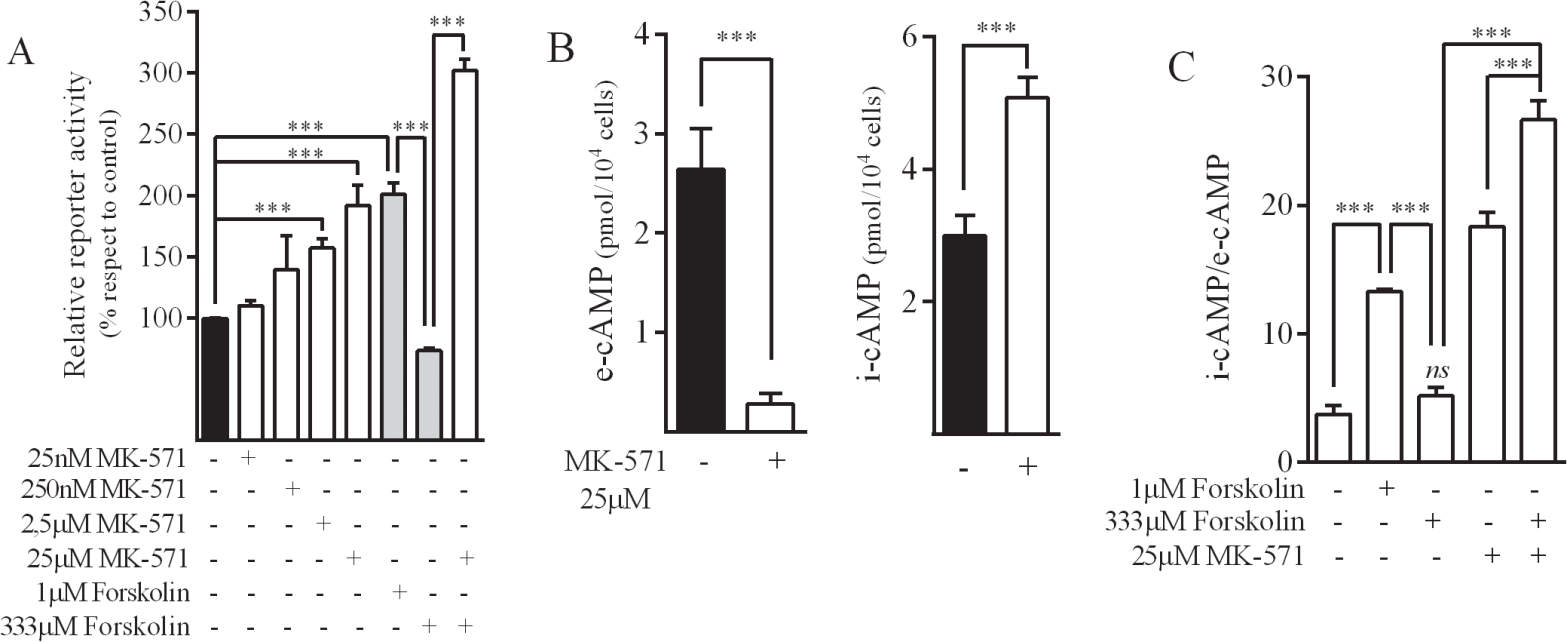
cAMP extrussion and regulation of MRP4 promoter in AR42J cells. ***A*.** *Effect of MK-571 (MRPs inhibitor) on MRP4 promoter activity*. Cells were transfected with MRP4-Luc and further exposed to the indicated agents. Luciferase activities were measured after 24h in the cell lysates and data was referred to untreated control. (mean±SD; n = 3), ***p<0.001. ***B*.** *Effect of MK-571 on extracellular (e-cAMP) and intracellular (i-cAMP) cAMP levels*. Cells were treated for 6h with MK-571 and cAMP levels were determined as described under Materials and Methods (mean±SD; n = 3). ***p<0.01. ***C*.** *e-cAMP/i-cAMP ratio*. Cells were treated for 10min with the indicated agents and cAMP levels were determined as described under Materials and Methods. The ratio between i-cAMP and e-cAMP values are shown as mean±SD; n = 3. ***p<0.01, *ns*. non-significant differences respect to basal.

In several cell types, including pancreatic acinar cells, MRP4 plays a crucial role in the physiological regulation of i-cAMP levels [29]. Therefore, we evaluated the effect of MRPs inhibition on MRP4 promoter activity by exposing AR42J cells to MK-571, a MRP inhibitor. Results showed that MK-571 treatment induced a concentration-dependent increase in luciferase activity (Fig. 6A). It is worth noting that when we evaluated cAMP modulation, MK-571 led to a 10-fold decrease in e-cAMP, and increased i-cAMP levels by 1.6-fold (Fig. 6B). Based on that, it seems like the balance between i-cAMP and e-cAMP levels could determine the effect of cAMP over MRP4 promoter activity. In this sense, high concentrations of forskolin (333μM) diminished MRP4 promoter activity, while low concentration of the agent (1μM) resulted in promoter activation (Fig. 6A). It is worth noting that i-cAMP/e-cAMP ratio after treatment with 333μM forskolin was similar to basal levels while in the case of 1μM forskolin, it was significantly higher than in untreated cells (Fig. 6C). Interestingly, when 333μM forskolin was added concomitantly with MK-571, both i-cAMP/e-cAMP ratio and luciferase activity were significantly higher than in control cells (Fig. 6A and C). These results suggest that cAMP increasing agents may induce MRP4 promoter activity only when i-cAMP/e-cAMP ratio achieved is significantly higher than basal. These findings could suggest that MRP4-mediated transport may be implicated in the regulation of MRP4 expression aside from the effects described for i-cAMP. In this regard, e-cAMP and/or another MRP4 endogenous substrate, such as PGE2, may also participate in MRP4 transcriptional regulation.

**Fig 7:**
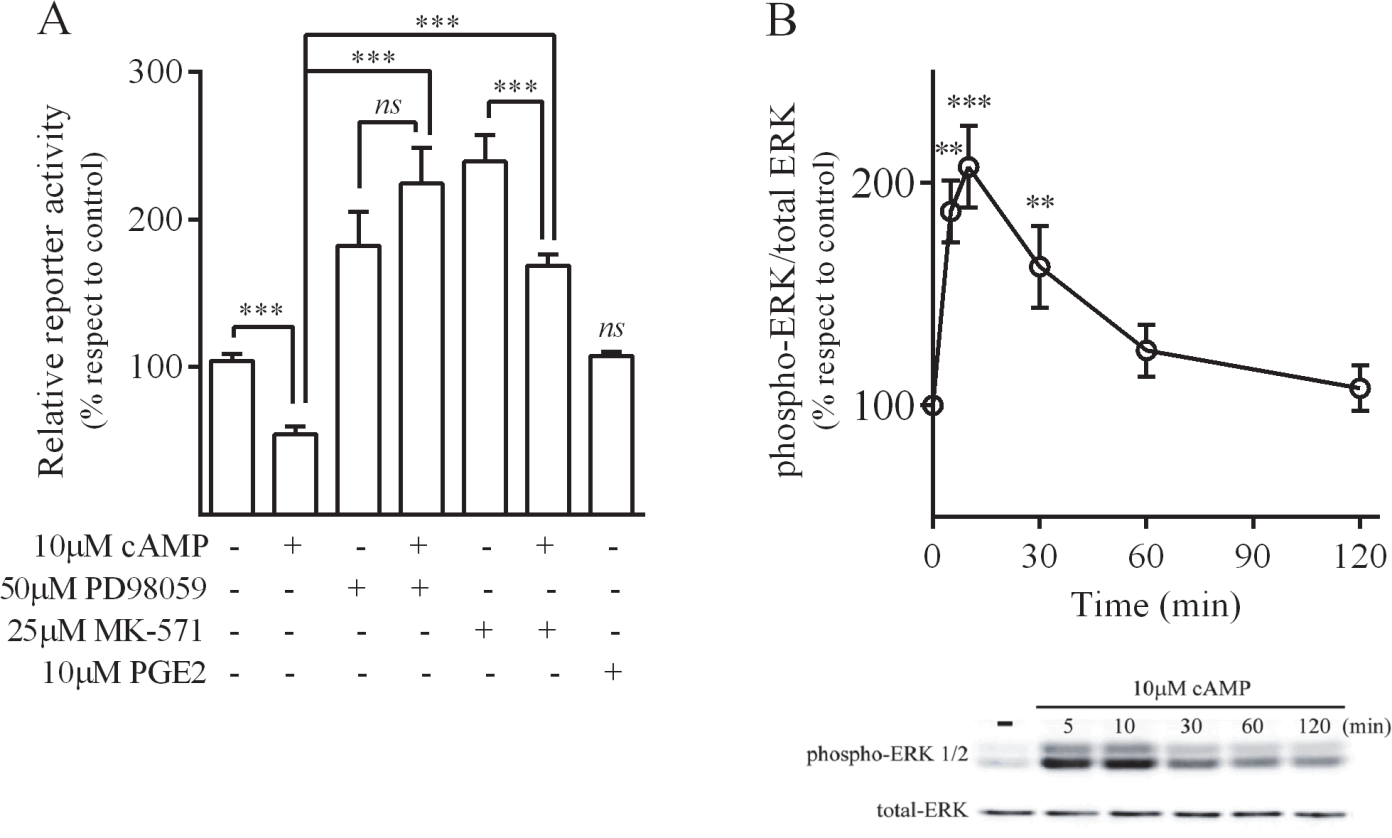
MEK/ERK involvement in the e-cAMP-mediated regulation of MRP4 promoter in AR42J cells. ***A*.** Cells were transfected with MRP4-Luc and further exposed to the indicated agents, luciferase activities were measured after 24h in cell lysates and referred to control (mean±SD; n = 3). ***B*.** *e-cAMP mediated ERK activation*. Cells were treated with 10mM cAMP at the time shown and p-ERK and total ERK levels were evaluated by western blot. *Left*. Densitometric quantification of p-ERK protein bands were normalized respect to total-ERK and referred to control (mean±SD; n = 3). *Right*. A representative western blot assay of three independent experiments is shown. ***p<0.001; **p<0.01; *ns*. non-significant difference respect to basal.

Previous reports proposed that extracellular cAMP may behave as a “third messenger” by mediating effects at the extracellular space [37]. Therefore, we addressed this possibility and observed that following 24h treatment with cAMP (which does not cross the plasma membrane), MRP4 promoter activity decreased by 45% suggesting that e-cAMP may also regulate MRP4 expression in a negative manner. When the effect of 10μM PGE2 was evaluated, it did not modify cAMP levels (data not shown) or luciferase activity in AR42J cells (Fig. 7A), excluding its involvement in the regulation of the MRP4 promoter observed after treatment with MK-571. Consistently, the addition of cAMP partially reversed MK-571 response (Fig. 7A).

Knowing that MEK/ERK pathway mediates a negative regulation over MRP4 promoter activity, we evaluated ERK involvement in the effects evoked by e-cAMP. Incubation of the cells with 10μM cAMP led to a rapid increase in phosphorylated ERK (P-ERK) levels, reaching a 2-fold increase after 10min treatment (Fig. 7B). The MEK/ERK inhibitor PD98059 completely abolished e-cAMP-mediated reduction in luciferase activity (Fig. 7A). These findings indicate that e-cAMP increase P-ERK levels in AR42J cells and reduced MRP4 promoter transcriptional activity through the MEK/ERK pathway. It has been reported that e-cAMP can be metabolized to adenosine by the action of ecto-phosphodiesterases and ecto-5´-nucleotidases, and that some products of this metabolic conversion are responsible for the observed effects of e-cAMP. Based on that, we cannot currently state whether the e-cAMP effect on the MRP4 promoter involves a direct or indirect mechanism and needs to be further investigated [37,38].

## Conclusion

The major finding of the present study was that MRP4 is transcriptionally regulated by cAMP through a mechanism where the balance between intracellular and extracellular cAMP plays a key role in the feedback regulation of the transporter expression. Sustained i-cAMP increase in AR42J cells induced the MRP4 promoter through the Epac/Rap1 pathway whereas e-cAMP inhibited it through ERK phosphorylation. Present findings may constitute one possible explanation to the higher expression of MRP4 observed in other pancreatic adenocarcinoma cells, as PANC1 cells, where exacerbated cAMP signaling has been described [30] respect to normal pancreatic tissue.

## Supporting Information

S1 Fig Analysis of MRP4 promoter. ***A*.** Analysis of putative transcription factor (TF) binding sites related to the cAMP signaling pathway using the Genomatrix Suite Software. The selected TF binding sites are indicated next to its position referred to the transcriptional starting site (TSS). ***B*.** Basal promoter activity of pGL3-Basic and MRP4-Luc constructs transfected in AR42J cells (mean±SD; n = 3), **p<0.01 respect to control.
(TIF)

## References

[1] Beavo JA, Brunton LL. Cyclic nucleotide research—still expanding after half a century. Nat Rev Mol Cell Biol. 2002;3: 710–718. 12209131 10.1038/nrm911

[2] Wielinga PR, van der Heijden I, Reid G, Beijnen JH, Wijnholds J, Borst P. Characterization of the MRP4- and MRP5-mediated transport of cyclic nucleotides from intact cells. J Biol Chem. 2003;278: 17664–17671. 12637526 10.1074/jbc.M212723200

[3] Chen ZS, Lee K, Kruh GD. Transport of cyclic nucleotides and estradiol 17-beta-D-glucuronide by multidrug resistance protein 4. Resistance to 6-mercaptopurine and 6-thioguanine. J Biol Chem. 2001;276: 33747–33754. 11447229 10.1074/jbc.M104833200

[4] van Aubel RA, Smeets PH, Peters JG, Bindels RJ, Russel FG. The MRP4/ABCC4 gene encodes a novel apical organic anion transporter in human kidney proximal tubules: putative efflux pump for urinary cAMP and cGMP. J Am Soc Nephrol. 2002;13: 595–603. 11856762 10.1681/ASN.V133595

[5] Reid G, Wielinga P, Zelcer N, van der Heijden I, Kuil A, de Haas M, et al The human multidrug resistance protein MRP4 functions as a prostaglandin efflux transporter and is inhibited by nonsteroidal antiinflammatory drugs. Proc Natl Acad Sci U S A. 2003;100: 9244–9249. 12835412 10.1073/pnas.1033060100 PMC170903

[6] Russel FG, Koenderink JB, Masereeuw R. Multidrug resistance protein 4 (MRP4/ABCC4): a versatile efflux transporter for drugs and signalling molecules. Trends Pharmacol Sci. 2008;29: 200–207. 10.1016/j.tips.2008.01.006 18353444

[7] Rius M, Nies AT, Hummel-Eisenbeiss J, Jedlitschky G, Keppler D. Cotransport of reduced glutathione with bile salts by MRP4 (ABCC4) localized to the basolateral hepatocyte membrane. Hepatology. 2003;38: 374–384. 12883481 10.1053/jhep.2003.50331

[8] Leggas M, Adachi M, Scheffer GL, Sun D, Wielinga P, Du G, et al Mrp4 confers resistance to topotecan and protects the brain from chemotherapy. Mol Cell Biol. 2004;24: 7612–7621. 15314169 10.1128/MCB.24.17.7612-7621.2004 PMC506999

[9] Ritter CA, Jedlitschky G, Meyer zu Schwabedissen H, Grube M, Kock K, Kroemer HK. Cellular export of drugs and signaling molecules by the ATP-binding cassette transporters MRP4 (ABCC4) and MRP5 (ABCC5). Drug Metab Rev. 2005;37: 253–278. 15747503 10.1081/dmr-200047984

[10] Chen ZS, Lee K, Walther S, Raftogianis RB, Kuwano M, Zeng H, et al Analysis of methotrexate and folate transport by multidrug resistance protein 4 (ABCC4): MRP4 is a component of the methotrexate efflux system. Cancer Res. 2002;62: 3144–3150. 12036927

[11] Reid G, Wielinga P, Zelcer N, De Haas M, Van Deemter L, Wijnholds J, et al Characterization of the transport of nucleoside analog drugs by the human multidrug resistance proteins MRP4 and MRP5. Mol Pharmacol. 2003;63: 1094–1103. 12695538 10.1124/mol.63.5.1094

[12] Rius M, Hummel-Eisenbeiss J, Hofmann AF, Keppler D. Substrate specificity of human ABCC4 (MRP4)-mediated cotransport of bile acids and reduced glutathione. Am J Physiol Gastrointest Liver Physiol. 2006;290: G640–649. 16282361 10.1152/ajpgi.00354.2005

[13] Zelcer N, Reid G, Wielinga P, Kuil A, van der Heijden I, Schuetz JD, et al Steroid and bile acid conjugates are substrates of human multidrug-resistance protein (MRP) 4 (ATP-binding cassette C4). Biochem J. 2003;371: 361–367. 12523936 10.1042/BJ20021886 PMC1223295

[14] Copsel S, Garcia C, Diez F, Vermeulem M, Baldi A, Bianciotti LG, et al Multidrug resistance protein 4 (MRP4/ABCC4) regulates cAMP cellular levels and controls human leukemia cell proliferation and differentiation. J Biol Chem. 2011;286: 6979–6988. 10.1074/jbc.M110.166868 21205825 PMC3044954

[15] Sun Y, Shi N, Lu H, Zhang J, Ma Y, Qiao Y, et al ABCC4 copy number variation is associated with susceptibility to esophageal squamous cell carcinoma. Carcinogenesis. 2014;35: 1941–1950. 10.1093/carcin/bgu043 24510239

[16] Qian Z, Zhu G, Tang L, Wang M, Zhang L, Fu j, et al Whole genome gene copy number profiling of gastric cancer identifies PAK1 and KRAS gene amplification as therapy targets. Genes Chromosomes Cancer. 2014;53: 883–894. 10.1002/gcc.22196 24935174

[17] Yu Z, Zhang C, Chai R, Du Y, Gao X, Xing J, et al Prognostic significance and molecular mechanism of ATP-binding cassette subfamily C member 4 in resistance to neoadjuvant radiotherapy of locally advanced rectal carcinoma. PLOS ONE. 2014;9: e85446 10.1371/journal.pone.0085446 24454870 PMC3893201

[18] Zhao X, Guo Y, Yue W, Zhang L, Gu M, Wang Y. ABCC4 is required for cell proliferation and tumorigenesis in non-small cell lung cancer. Onco Targets Ther. 2014;7: 343–351. 10.2147/OTT.S56029 24591841 PMC3937249

[19] Bagnoli M, Beretta GL, Gatti L, Pilotti S, Alberti P, Tarantino E, et al Clinicopathological impact of ABCC1/MRP1 and ABCC4/MRP4 in epithelial ovarian carcinoma. Biomed Res Int. 2013;2013: 143202 10.1155/2013/143202 24024181 PMC3760178

[20] Montani M, Hermanns T, Muntener M, Wild P, Sulser T, Kristiansen G. Multidrug resistance protein 4 (MRP4) expression in prostate cancer is associated with androgen signaling and decreases with tumor progression. Virchows Arch. 2013;462: 437–443. 10.1007/s00428-013-1390-8 23503867

[21] Norris MD, Smith J, Tanabe K, Tobin P, Flemming C, Scheffer GL, et al Expression of multidrug transporter MRP4/ABCC4 is a marker of poor prognosis in neuroblastoma and confers resistance to irinotecan in vitro. Mol Cancer Ther. 2005;4: 547–553. 15827327 10.1158/1535-7163.MCT-04-0161

[22] Huynh T, Norris MD, Haber M, Henderson MJ. ABCC4/MRP4: a MYCN-regulated transporter and potential therapeutic target in neuroblastoma. Front Oncol. 2012;2: 178 10.3389/fonc.2012.00178 23267433 PMC3526013

[23] Rahib L, Smith BD, Aizenberg R, Rosenzweig AB, Fleshman JM, Matrisian LM. Projecting cancer incidence and deaths to 2030: the unexpected burden of thyroid, liver, and pancreas cancers in the United States. Cancer Res. 2014;74: 2913–2921. 10.1158/0008-5472.CAN-14-0155 24840647

[24] Lohr M. Is it possible to survive pancreatic cancer? Nat Clin Pract Gastroenterol Hepatol. 2006;3: 236–237. 16672986 10.1038/ncpgasthep0469

[25] Zhang Z, Wang J, Shen B, Peng C, Zheng M. The ABCC4 gene is a promising target for pancreatic cancer therapy. Gene. 2012;491: 194–199. 10.1016/j.gene.2011.09.029 21989485

[26] Gu X, Manautou JE. Regulation of hepatic ABCC transporters by xenobiotics and in disease states. Drug Metab Rev. 2010;42: 482–538. 10.3109/03602531003654915 20233023 PMC4458072

[27] Davio CA, Cricco GP, Bergoc RM, Rivera ES. H1 and H2 histamine receptors in N-nitroso-N-methylurea (NMU)-induced carcinomas with atypical coupling to signal transducers. Biochem Pharmacol. 1995;50: 91–96. 7605350 10.1016/0006-2952(95)00108-c

[28] Hochbaum D, Tanos T, Ribeiro-Neto F, Altschuler D, Coso OA. Activation of JNK by Epac is independent of its activity as a Rap guanine nucleotide exchanger. J Biol Chem. 2003;278: 33738–33746. 12783872 10.1074/jbc.M305208200

[29] Rodriguez MR, Diez F, Ventimiglia MS, Morales V, Copsel S, Vatta MS, et al Atrial natriuretic factor stimulates efflux of cAMP in rat exocrine pancreas via multidrug resistance-associated proteins. Gastroenterology. 2011;140: 1292–1302. 10.1053/j.gastro.2010.12.053 21237168

[30] O'Hayre M, Vazquez-Prado J, Kufareva I, Stawiski EW, Handel TM, Seshagiri S, et al The emerging mutational landscape of G proteins and G-protein-coupled receptors in cancer. Nat Rev Cancer. 2013;13: 412–424. 10.1038/nrc3521 23640210 PMC4068741

[31] Gloerich M, Bos JL. Epac: defining a new mechanism for cAMP action. Annu Rev Pharmacol Toxicol. 2010;50: 355–375. 10.1146/annurev.pharmtox.010909.105714 20055708

[32] Breckler M, Berthouze M, Laurent AC, Crozatier B, Morel E, Lezoualc´h F. Rap-linked cAMP signaling Epac proteins: compartmentation, functioning and disease implications. Cell Signal. 2011;23: 1257–1266. 10.1016/j.cellsig.2011.03.007 21402149

[33] Broderdorf S, Zang S, Schaletzki Y, Grube M, Kroemer HK, Jedlitschky G. cAMP regulates expression of the cyclic nucleotide transporter MRP4 (ABCC4) through the EPAC pathway. Pharmacogenet Genomics. 2014;24: 522–526. 10.1097/FPC.0000000000000084 25121519

[34] Dreuw A, Hermanns HM, Heise R, Joussen S, Rodriguez F, Marquardt Y, et al Interleukin-6-type cytokines upregulate expression of multidrug resistance-associated proteins in NHEK and dermal fibroblasts. J Invest Dermatol. 2005;124: 28–37. 15654950 10.1111/j.0022-202X.2004.23499.x

[35] Stork PJ, Schmitt JM. Crosstalk between cAMP and MAP kinase signaling in the regulation of cell proliferation. Trends Cell Biol. 2002;12: 258–266. 12074885 10.1016/s0962-8924(02)02294-8

[36] Burgun C, Esteve L, Humblot N, Aunis D, Zwiller J. Cyclic AMP-elevating agents induce the expression of MAP kinase phosphatase-1 in PC12 cells. FEBS Lett. 2000;484: 189–193. 11078876 10.1016/s0014-5793(00)02153-0

[37] Hofer AM, Lefkimmiatis K. Extracellular calcium and cAMP: second messengers as "third messengers"? Physiology (Bethesda). 2007;22: 320–327. 17928545 10.1152/physiol.00019.2007

[38] Osycka-Salut C, Diez F, Burdet J, Gervasi MG, Franchi A, Bianciotti LG, et al Cyclic AMP efflux, via MRPs and A1 adenosine receptors, is critical for bovine sperm capacitation. Mol Hum Reprod. 2014;20: 89–99. 10.1093/molehr/gat053 23907162

