# Supplementary figures and images for: Dual Role of cAMP in the Transcriptional Regulation of Multidrug Resistance-Associated Protein 4 (MRP4) in Pancreatic Adenocarcinoma Cell Lines
Source: PLoS One. 2015 Mar 19;10(3):e0120651. doi: 10.1371/journal.pone.0120651 (PMC4366062; doi:10.1371/journal.pone.0120651)

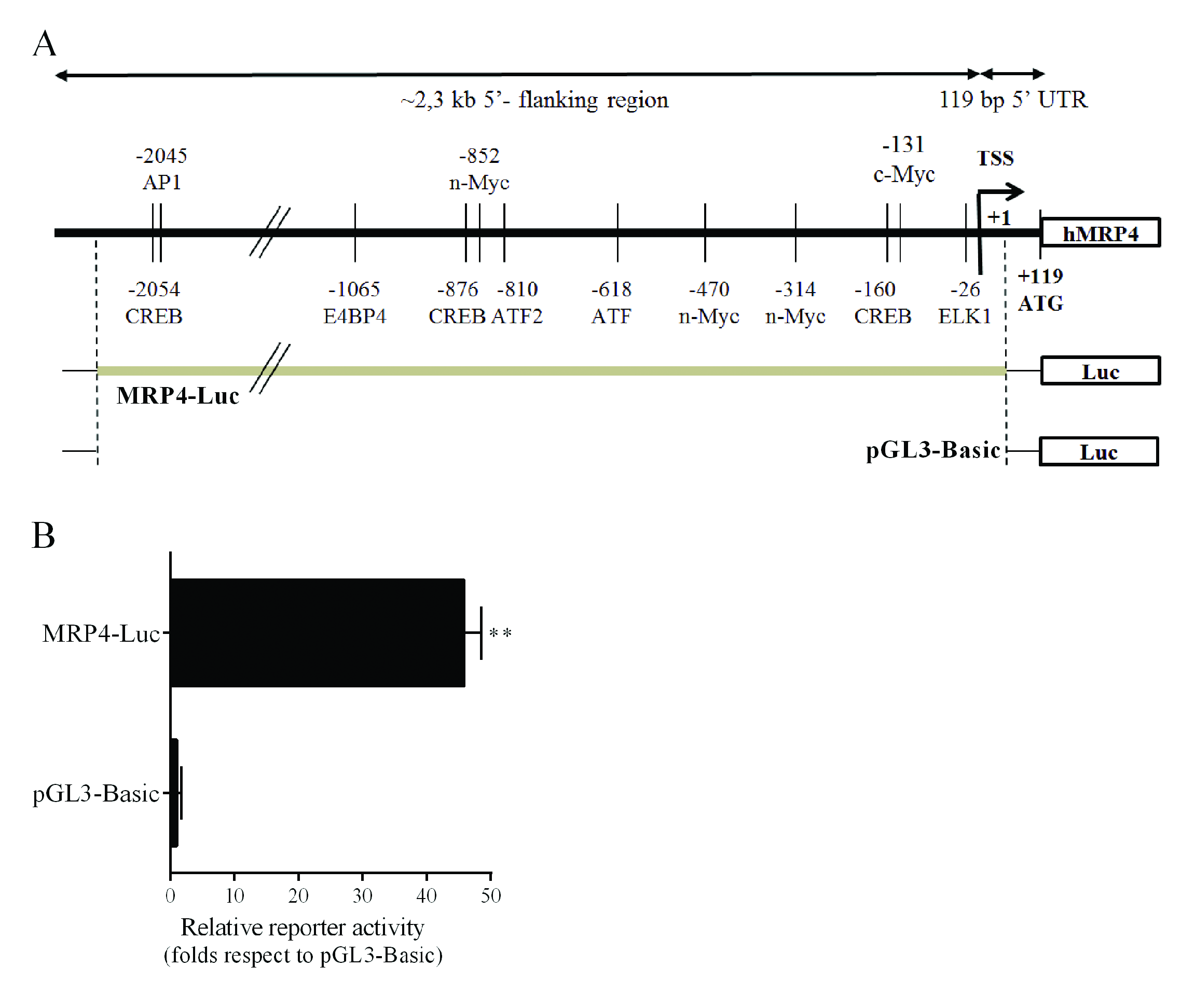

Supplement: S1 Fig — A. Analysis of putative transcription factor (TF) binding sites related to the cAMP signaling pathway using the Genomatrix Suite Software. The selected TF binding sites are indicated next to its position referred to the transcriptional starting site (TSS). B. Basal promoter activity of pGL3-Basic and MRP4-Luc constructs transfected in AR42J cells (mean±SD; n = 3), **p<0.01 respect to control. (TIF) [file pone.0120651.s001.tif]
